# Pulmonary Artery Smooth Muscle Cell Senescence Promotes the Proliferation of PASMCs by Paracrine IL-6 in Hypoxia-Induced Pulmonary Hypertension

**DOI:** 10.3389/fphys.2021.656139

**Published:** 2021-04-07

**Authors:** Ai-Ping Wang, Fang Yang, Ying Tian, Jian-Hui Su, Qing Gu, Wei Chen, Shao-Xin Gong, Xiao-Feng Ma, Xu-Ping Qin, Zhi-Sheng Jiang

**Affiliations:** ^1^Institute of Cardiovascular Disease, Key Lab for Arteriosclerology of Hunan Province, University of South China, Hengyang, China; ^2^Department of Physiology, Institute of Neuroscience, Hengyang Key Laboratory of Neurodegeneration and Cognitive Impairment, Hengyang Medical College, University of South China, Hengyang, China; ^3^Institute of Clinical Research, Affiliated Nanhua Hospital, University of South China, Hengyang, China; ^4^Laboratory of Vascular Biology, Institute of Pharmacy and Pharmacology, University of South China, Hengyang, China; ^5^State Key Laboratory of Cardiovascular Disease, Fuwai Hospital, National Center for Cardiovascular Diseases, Chinese Academy of Medical Sciences and Peking Union Medical College, Beijing, China; ^6^Department of Pathology, The First Affiliated Hospital, University of South China, Hengyang, China

**Keywords:** pulmonary artery smooth muscle cells senescence, interleukin-6 (IL-6), mTOR- S6K1 signaling, pulmonary hypertension, proliferation

## Abstract

Pulmonary hypertension (PH) is a critical and dangerous disease in cardiovascular system. Pulmonary vascular remodeling is an important pathophysiological mechanism for the development of pulmonary arterial hypertension. Pulmonary artery smooth muscle cell (PASMC) proliferation, hypertrophy, and enhancing secretory activity are the main causes of pulmonary vascular remodeling. Previous studies have proven that various active substances and inflammatory factors, such as interleukin 6 (IL-6), IL-8, chemotactic factor for monocyte 1, etc., are involved in pulmonary vascular remodeling in PH. However, the underlying mechanisms of these active substances to promote the PASMC proliferation remain to be elucidated. In our study, we demonstrated that PASMC senescence, as a physiopathologic mechanism, played an essential role in hypoxia-induced PASMC proliferation. In the progression of PH, senescence PASMCs could contribute to PASMC proliferation via increasing the expression of paracrine IL-6 (senescence-associated secretory phenotype). In addition, we found that activated mTOR/S6K1 pathway can promote PASMC senescence and elevate hypoxia-induced PASMC proliferation. Further study revealed that the activation of mTOR/S6K1 pathway was responsible for senescence PASMCs inducing PASMC proliferation via paracrine IL-6. Targeted inhibition of PASMC senescence could effectively suppress PASMC proliferation and relieve pulmonary vascular remodeling in PH, indicating a potential for the exploration of novel anti-PH strategies.

## Introduction

Pulmonary hypertension (PH) is a malignant disease of cardiovascular system caused by pulmonary arteriole vascular hyperplasia, which leads to the progressive increase of pulmonary vascular resistance and the increase of right ventricular load and finally leads to right heart failure (Lan et al., 2018). Pulmonary vascular remodeling is the key pathophysiological mechanism of the occurrence and development of PH (Shah, 2012; Maron et al., 2020). Pulmonary vascular remodeling involves a variety of cells, including vascular endothelial cells, pulmonary artery smooth muscle cells (PASMCs), and multiple inflammatory cells. However, proliferation, hypertrophy, and increased secretory activity of PASMCs are the main pathological changes of pulmonary vascular remodeling in PH. In recent years, new drugs for the treatment of PH have been found for different targets, such as soluble ornithine cyclase agonists, tyrosine kinase inhibitors, and so on. Although these drugs have a certain effect on improving the clinical symptoms of patients with PH, they still cannot effectively reduce the mortality (Macchia et al., 2014; Samokhin et al., 2020). The reason is that the pathogenesis of the disease has not been fully clarified (Tang et al., 2016; Frid et al., 2020). Therefore, in-depth study of the pathogenesis of pulmonary vascular remodeling in PH is of great significance for finding and confirming targets and developing new drugs for the treatment of PH.

Cell senescence is a process characterized by irreversible loss of cell growth and proliferation, which is mainly characterized by the cessation of cell replication, cell cycle arrest in G_0/_G_1_ phase, telomere shortening, up-regulation of senescence-related genes, and significant increase of specific P-galactose activity (Leon and Gustafsson, 2016; van der Feen et al., 2019). It is reported that cell senescence is closely related to the occurrence and development of many human diseases (Wang et al., 2015; Leon and Gustafsson, 2016). Previous studies have shown that senescent cells eventually lead to programmed death apoptosis (Hoenicke and Zender, 2012). Recent studies have found that senescent cells can show “senescence but not aging”; senescent cells can promote the occurrence and development of disease by secreting cytokine senescence–associated secretory phenotype (SASP). For example, SASP secreted by aging fibroblasts can promote the epithelial–mesenchymal transition of epithelial cancer cells (Coppe et al., 2010) and improve its tumorigenic potential (Krtolica et al., 2001). However, the role of vascular smooth muscle cells senescence in the proliferation of PASMCs in PH remains unclear.

It has been found that endothelial cell senescence is involved in the formation and development of atherosclerosis, diabetes, vascular remodeling and inflammation, blood test blockage, and so on (Wang and Bennett, 2012; Kim et al., 2020). In recent years, studies have proved that the senescence and phenotypic changes of vascular smooth muscle cells also play an important role in the occurrence and development of cardiovascular diseases (Song et al., 2012; Gardner et al., 2015). For example, vascular smooth muscle cells senescence can promote atherosclerosis and plaque vulnerability (Rabinovitch, 2012); senescence can also up-regulate the secretion of interleukin 6 (IL-6) and CCL2 production in aortic smooth muscle cells, causing chronic inflammation and promoting atherosclerosis (Song et al., 2012). It is worth noting that recent studies have found that PASMC senescence is significantly increased in (pulmonary vascular remodeling in chronic obstructive pulmonary disease (COPD) (Noureddine et al., 2011). In our pre-experiment, we found that after hypoxia treatment, the number of β-galactosidase–positive cells in PASMCs increased significantly, and the expression of senescence index p21 was significantly up-regulated. Therefore, we speculate that PASMC senescence may play an important role in hypoxia-induced PASMC proliferation and PH.

As mentioned previously, PASMC senescence plays an important role in hypoxia-induced pulmonary vascular remodeling, so what is the mechanism by which aging PASMCs promote PASMC cell proliferation and pulmonary vascular remodeling? It has been reported that senescent cells can produce a variety of cytokines (SASP), including IL-6, IL-8, tumor necrosis factor α (TNF-α), monocyte chemoattractant protein 1 (MCP-1), etc. (Coppe et al., 2010; Acosta et al., 2013), which promotes the occurrence and development of tumor; paracrine IL-6 can induce the proliferation of human lung fibroblasts and the growth and metastasis of pancreatic cancer cells (Huang et al., 2016; Klee et al., 2016). In addition, Gardner and colleagues have shown that aging aortic smooth muscle cells promote the occurrence and development of atherosclerosis by secreting SASP (IL-l α) (Gardner et al., 2015). These results suggest that senescent smooth muscle cells can secrete SASP and play an important role in the process of cardiovascular disease. Studies have confirmed that neutralizing antibodies against IL-6 can inhibit the proliferation of HepG2 cells (Hu et al., 2017). In addition, it was found that miR-195 could reduce the levels of IL-1 β, IL-6, and IL-8 and then inhibit the proliferation, migration, and intimal hyperplasia of vascular smooth muscle cells (Wang et al., 2012). More importantly, senescence PASMCs in COPD promote PASMC proliferation and pulmonary vascular remodeling by paracrine IL-6 (Noureddine et al., 2011). PASMCs senescence is markedly an increase in hypoxia-induced PH and senescence PASMCs can promote the proliferation of PASMCs by secreting SASP. And the senescence of PASMCs is mainly secreting SASP in which cytokines are involved in regulating the proliferation of PASMCs. In order to confirm which cytokines are mainly involved in the proliferation of PASMCs, we examined the expression of IL-1β, transforming growth factor β (TGF-β), IL-6, IL-8, MCP-1, and TNF-α in the culture medium of PASMC senescence induced by hypoxia. The results of pre-experiment showed that the expression of IL-6 in hypoxia-induced PASMC senescence was significantly increased. The results suggest that IL-6 may play a more important role than other cytokines. Therefore, we speculate that hypoxia can induce the senescence of PASMCs, and senescence of PASMCs may promote the abnormal proliferation of PASMCs by paracrine IL-6.

## Materials and Methods

### Animals

Male Sprague–Dawley (SD) rats (weighing 180–220 g) were obtained from the Laboratory Animal Center, University of South China (Hengyang, China). All surviving animals were handled in accordance with the National Institutes of Health Guide for the Care and Use of Laboratory Animals. The experimental protocol was approved by the medicine animal welfare committee of University of South China (Hengyang, China).

### Separation, Culture, and Identification of PASMCs in Rats

The primary PASMCs in SD rats are cultured by tissue walling method; 110–150 g male SD rat was anesthetized by intraperitoneal injection of 10% chloral hydrate (3 mL kg^–1^). The pulmonary artery was quickly taken out from the SD rat; the outer membrane of blood vessel was gently removed; the intima was removed with ophthalmic forceps after longitudinal cutting, cut into 1 × 1-mm^3^ fragments, and was seeded in 25-cm^2^ flasks; the growth of cells was observed by inverted microscope after 4 to 5 days of culture in a complete incubator [20% fetal bovine serum (FBS)–Dulbecco modified Eagle medium (DMEM)] and 1% antibiotic (penicillin–streptomycin). The tissue block was carefully removed with dental probe when the cells around the tissue block were almost full (about 6 days), and culture was continued for 1–3 days, at 37°C under 5% CO_2_ in DMEM containing 20% FBS. The primary PASMCs were observed under inverted microscope. The cells grew in a long spindle shape with abundant cytoplasm; when they grew compactly, they were arranged in parallel bundles and distributed in a characteristic “peak valley” shape of smooth muscle cells. The cells were then counted and seeded (passage 2) in 75-cm^2^ flasks. Purified PASMCs were screened by 0.25% pancreatic enzyme digestion and gradient digestion. The third-generation cells were identified by immunohistochemistry staining using an antibody against smooth muscle α-actin (ab7817, 1:50; Abcam, Hong Kong, China). The purity of the PASMCs can reach more than 96%. PASMCs between passage 3 to 10 were used for the next experiments.

### Establishing of Hypoxia-Induced PASMC Proliferation

All experimental animal procedures were conducted in accordance with human animal care standards with approval from the University of South China Ethics Committee (Hengyang, China). The animals were acclimated to the environment for 1 week prior to treatment under specific pathogen-free conditions. The PASMCs were cultured with DMEM medium of 10% FBS and treated with DMEM medium of 1% FBS for 24 h when the cells cultured to confluence for 75%. In the following experiment, the normoxic group was cultured in incubator of 37°C, 5% CO_2_. The hypoxia group (3% O_2_) was placed in a small hypoxia chamber. Hypoxia significantly induced PASMC proliferation by CCK8 kit, and 48-h proliferation range reached the maximum; therefore, the 48 h was selected as the optimized time point for subsequent experiments. The small hypoxia chamber (Changjin, China) consists of 92% N_2_, 5% CO_2_, and 3% O_2_.

### Detection of PASMC Senescence by β-Galactosidase Staining Kit

The cell senescence β-galactosidase staining kit (Beyotime Biotechnology, Shanghai, China) was used to detect the level of senescence-associated β-galactosidase activity in hypoxia-induced PASMC proliferation. SA-β-Gal staining was performed according to the manufacturer’s instructions. Under an optical microscope, it can be observed that senescence cells are dyed into blue in hypoxia-induced PASMC proliferation. Briefly, cells were placed in a 3% small hypoxia compartment for 48 h. Next, cells were washed twice in phosphate-buffered saline (PBS); staining solution was added to the PASMCs for 15 min at room temperature. Then cells were washed with PBS for three times and stained with working solution overnight at a 37°C electrothermal incubator.

### Western Blot

For protein extraction, isolated PASMCs were washed with PBS and lysed with RIPA lysis buffer. RIPA lysis buffer and phenylmethylsulphonyl fluoride (9:1) were used to extract protein. Protein contents were measured by BCA protein assay kit. In addition, separation of proteins by size was performed using sodium dodecyl sulfate–polyacrylamide gel by electrophoresis. After electrophoresis, proteins from the gel were transferred onto polyvinylidene difluoride (Billerica, MA, United States) membranes (Millipore). After transfer, the objective proteins membranes were saturated with 5% milk for 2 h. The objective proteins membrane then incubated overnight at 4°C with specific primary antibodies: p21 (1:2,000 dilution) and PCNA (1:2,000 dilution) were purchased from Abcam (Cambridge, United Kingdom); PI3K rabbit antibody (1:1,000 dilution) and m-TOR (1:1,000 dilution) rabbit monoclonal antibody were obtained from Cell Signaling Technology (Danvers, MA, United States); GAPDH (1:5,000 dilution) and β-actin (1:5,000 dilution) mouse antibody were from Proteintech (Shanghai, China). Horseradish peroxidase–conjugated anti-rabbit or anti-mouse immunoglobulin G was used to amplify the signals from the primary antibody. The densitometry analysis of objective protein bands was performed using software (Alphalmager TM 2200). Protein expression was reported as the protein/β-actin ratio and expressed as arbitrary units.

### PASMC Proliferation Detection by CCK-8

Pulmonary artery smooth muscle cell proliferation was measured by Cell Counting Kit-8 (CCK-8) assay (Beyotime, C0038, China) according to the manufacturer’s instructions. Cell Counting Kit-8 (CCK-8) is a water-soluble tetrazolium salt, which is reduced to orange-yellow xanthium by some dehydrogenase in mitochondria in the presence of 1-Methoxyphenazine methosulfate. The darker the cell color, the more the number of methylinders produced, and the more the number of living cells. The number of living cells can be indirectly reflected by the light absorption value measured at 450-nm wavelength by enzyme-labeled instrument. Briefly, the cells were seeded onto 96-well dishes. The normoxic group cells were seeded in incubator of 37°C, 5% CO_2_; the hypoxia group (3% O_2_) cells were cultured in a small hypoxia chamber for 48 h in 10% fetal bovine serum (Grand Island, NY, United States) before 6 h in 0.1% FBS. After 48 h, the medium was changed to the same medium supplemented with the CCK8 liquid according to 10% of the total volume of each well medium, and then the cells were incubated for 1–2 h, and the absorbance was detected at the wave length of 450 nm using an enzyme labeling instrument.

### Measurements of IL-6 by Enzyme-Linked Immunosorbent Assay

Soluble factors were measured in cell medium. The main principle of IL-6 enzyme linked immunosorbent assay (ELISA) Kit (BOSTER, EK0412) is that the substrate TMB is catalyzed by peroxidase to blue, which changes from blue to yellow under the action of acid. The level of IL-6 in the sample is positively correlated with the depth of color. For cell medium determinations, PASMCs from early and late passages were grown to confluence in DMEM containing 20% fetal calf serum. The medium was then removed, and the cells subjected to growth arrest. After 48 h of incubation, the conditional medium was used for quantitation of IL-6 using Quantikine ELISA kits according to the manufacturer’s instructions. The OD value was measured at 450 nm in the microplate reader.

### RNA Concentration Determination and Quality Evaluation

The RNA concentration was determined by biological spectrophotometer, the sample type was selected RNA, the determination system was 2 μL RNA sample solution, and the blank correction was carried out with 2 μL DEPC water before determination. OD260/230, OD260/280, RNA concentration, and A320 were recorded to evaluate RNA quality (the optimum range of A260/A280 is 1.8–2.0; less than 1.8 indicates that the contamination of protein or other organic matter in the solution is obvious; more than 2.2 indicates that the RNA may be degraded; the A320 value represents the number of salt ions in the RNA, and the general value less than 0.01 indicates that the saline ions content is very small).

### mRNA Reverse Transcription

Total RNA was extracted from cultured cells using Trizol reagent (Invitrogen, Carlsbad, CA, United States). For detection of mRNA expression, RNA (0.2–0.5 μg) was subjected to reverse transcription reaction using the PrimeScript reverse transcription reagent Kit (DRR037A; TaKaRa, Dalian, China) according to Takara Company’s reverse transcription kit. The RNA reverse transcription was carried out by the one-step method.

### Real-Time Polymerase Chain Reaction

Quantitative analysis of mRNA expression was performed using SYBR Premix Ex Taq (DRR420A; TaKaRa) at ABI 7300 system (7300 Real-time PCR instrument, ABI Company). The target gene fragment was amplified by PCR reaction on the fluorescence real-time quantitative polymerase chain reaction (qPCR) instrument. PCR cycling conditions were as follows: an initial incubation at 95°C for 15 s, followed by 40 cycles of denaturation at 95°C for 5 s, and annealing at 60°C for 31 s.

### Rapamycin of mTOR Inhibitor–Treated PASMCs

After taking the logarithmic growth stage cell seed plate, the fusion rate reached 70–80%, the low serum was synchronized for 24 h, and then the target cells were treated with different concentrations of rapamycin: normoxic group (normoxia): cell normal oxygen partial pressure (21% O_2_) treatment; hypoxia model group (hypoxia): cells were treated with 3% O_2_; hypoxia + 20 nM rapamycin group: the cells were added 20 nM rapamycin and cultured in hypoxia incubator for 48 h; hypoxia + 40 nM rapamycin group: the cells were added 40 nM rapamycin and cultured in hypoxia incubator for 48 h; and hypoxia + 80 nM rapamycin group: the cells were added 80 nM rapamycin and cultured in hypoxia incubator for 48 h. After the treatment, the expression of related proteins was collected according to the impassable experimental purpose.

### Small Interference RNA Transfection

The mTOR siRNA was purchased from RiboBio (Guangzhou, China). TurboFect transfection reagent (#0531; Thermo Scientific, United States) was used for cell transfection. The mTOR siRNA interference was transiently transfected. When PASMC growth reaches 60–70% fusion, the low serum medium was used for synchronization for 24 h. According to the experimental groups, a certain volume of serum-free culture medium and unrelated fragment transfection complex was added into the cells at 37°C, 5% CO_2_ for 4–6 h and then was replaced by 20% fetal bovine serum and cultured for 48 h. The experiment was divided into six groups: normoxia group (normoxia): normal oxygen partial pressure (21% O_2_) treatment; hypoxia group (hypoxia): cells were treated with 3% O_2_; hypoxia + negative group: the cells were transfected with negative control (50 nM) for 4–6 h and then subjected to hypoxia for 48 h; hypoxia + siRNA (25 nM) group: the cells were transfected with siRNA (25 nM) for 4–6 h and then subjected to hypoxia for 48 h; hypoxia + siRNA (50 nM) group: the cells were transfected with siRNA (50 nM) for 4–6 h and then subjected to hypoxia for 48 h; and hypoxia + siRNA (100 nM) group: the cells were transfected with siRNA (100 nM) for 4–6 h and then subjected to hypoxia for 48 h. Transfection efficiency was evaluated by mTOR mRNA and protein expression using real-time PCR and Western blot analysis, respectively. Each experiment was repeated using primarily cultured cells (3–10 passages). The experiments were repeated three times.

### Statistical Analysis

Statistical tests and graphs were done with SPSS 20.0 and GraphPad Prism 6.0. Values are expressed as fold change or mean ± standard error of the mean. Unpaired Student *t* tests were used for comparisons between two groups, and one-way analysis of variance was used for multiple groups (SPSS 20.0, Chicago, IL, United States). *P* < 0.05 was considered statistically significant.

## Results

### Increased PASMC Senescence Occurred in Hypoxia-Induced PASMC Proliferation

**FIGURE 1:**
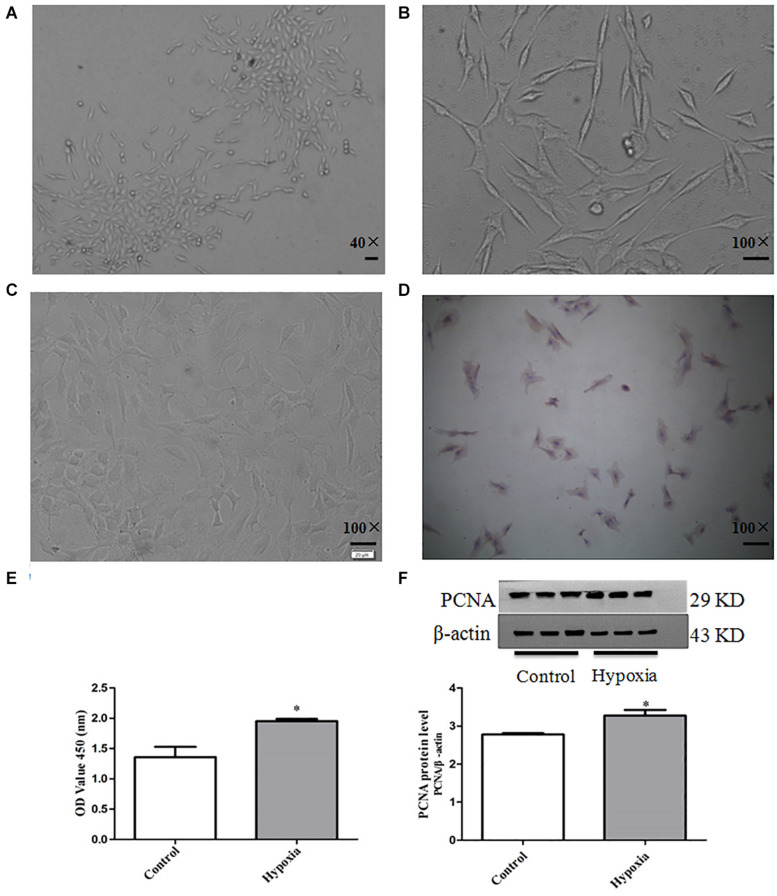
The culture and identification of primary PASMCs and establishment of PASMC proliferation model. **(A)** Cell morphology of third passage (40×), scale bar = 40 mm. **(B)** Cell morphology of sixth passage (100×), scale bar = 100 mm. **(C)** Negative control (100×), scale bar = 100 mm. **(D)** α-Smooth muscle actin immunohistochemistry (100×), scale bar = 100 mm. **(E)** The cell viability of hypoxia-induced PASMC proliferation by CCK8 for 48 h. **(F)** The expression of proliferative marker protein PCNA in PASMCs for 48 h. Control: PASMCs were cultured in 37°C, 5% CO_2_ incubator; hypoxia: 3% O_2_, PASMCs were cultured in small hypoxia chamber for 48 h. Data are mean ± SEM, *n* = 3. *P < 0.05 vs. control.

It can be observed that the primary PASMCs show adherent growth; the cell morphology is mostly fusiform and abundant cytoplasm, which is the characteristic “peak valley” distribution using inverted phase contrast microscope after culturing for 5–7 days. Figures 1A,B show passages 3 and 6 PASMCs, respectively. α-Smooth muscle actin immunohistochemical showed that the positive stained rate of PASMCs was more than 96%, and the purity was greater than 96% (Figures 1C,D).

The protein expression of PCNA was detected by Western blot, and the proliferation of PASMCs was detected by CCK8 assay. The result of CCK8 showed that the proliferation of PASMCs in hypoxia treatment group was higher than that of normoxic group (*P* < 0.05). The results of Western blot showed that the protein expression PCNA in hypoxia treatment group was significantly up-regulated compared with that in the normoxic group (*P* < 0.05) (Figures 1E,F). These results show that hypoxia can significantly promote PASMC proliferation.

**FIGURE 2:**
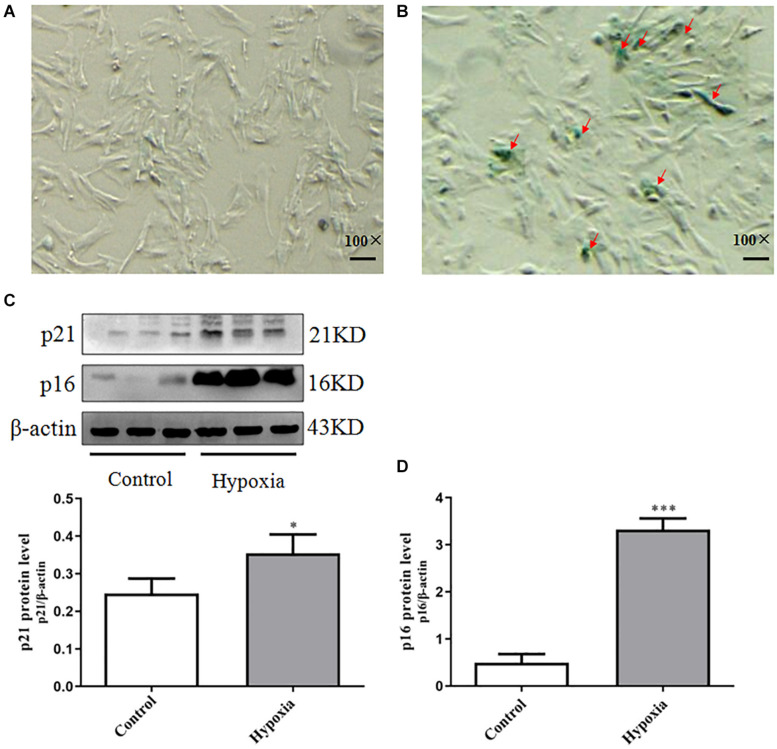
Increased PASMC senescence occurred in hypoxia-induced PASMC proliferation. **(A,B)** The positive expression of SA-β-Gal staining in hypoxia-induced PASMC proliferation was measured by SA-β-Gal staining. **(A)** Control (100×), scale bar = 100 mm. **(B)** Hypoxia (100×), scale bar = 100 mm. **(C)** The expression of p21 in hypoxia-induced PASMC proliferation for 48 h. **(D)** The expression of p16 in hypoxia-induced PASMC proliferation for 48 h. Control: PASMCs were cultured in 37°C, 5% CO_2_ incubator; hypoxia: 3% O_2_, PASMCs were cultured in small hypoxia chamber for 48 h. Data are mean ± SEM, *n* = 3. ^∗^*P* < 0.05, ^∗∗^*P* < 0.01 vs. control, ^∗∗∗^*P* < 0.001 vs. control.

In order to confirm whether PASMCs were senescent in hypoxia-induced PASMC proliferation, we performed SA-β-Gal staining in hypoxia-induced PASMC proliferation. We founded that positive staining of SA-β-Gal was markedly increased in hypoxia-induced PASMC proliferation (Figures 2A,B). These results suggest that PASMC senescence is markedly increased in hypoxia-treated PASMCs.

Further testing the expression of senescence markers p21 and p16 in hypoxia-induced PASMC proliferation, we found that p21 and p16 were elevated in hypoxia-treated PASMCs, indicating that they were senescent PASMCs (Figures 2C,D). These results demonstrated PASMC senescence may be tightly correlated with PASMC proliferation in Hypoxia-induced pulmonary hypertension (HPH).

### Senescent PASMCs Promote PASMC Proliferation by Paracrine IL-6

**FIGURE 3:**
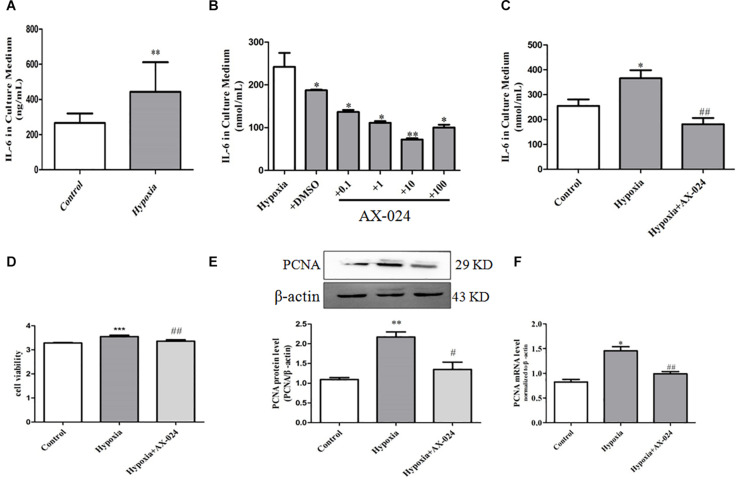
Senescent PASMCs promote PASMC proliferation by paracrine IL-6 and AX-024 of IL-6 inhibitor can inhibit cell proliferation by inhibiting paracrine IL-6 in hypoxia-treated PASMCs. **(A)** The paracrine level of IL-6 in the supernatant in PASMCs by ELISA kit. **(B)** The level of IL-6 in the culture medium of hypoxia-induced PASMC proliferation by different concentrations of AX-024. **(C)** The level of IL-6 in the culture medium in different groups in PASMCs after AX-024 treatment, Proliferation of PASMCs was measured by CCK8 assay after incubation with different AX-024 (0.01, 0.1, 1, 10, 100, 1,000 nmol/mL). **(D)** The cell viability detection in PASMCs by CCK8 after treating with different concentration of AX-024. **(E)** The protein expression of PCNA was measured by Western blot. **(F)** The mRNA expression of PCNA was measured by qPCR. Data are mean ± SEM, *n* = 3. ^∗^*P* < 0.05, ^∗∗^*P* < 0.01 vs. control, ^∗∗∗^*P* < 0.001 vs. control, ^#^*P* < 0.05 vs. hypoxia, ^##^*P* < 0.01 vs. hypoxia; NS indicates no significance.

In order to further confirm the role of PASMC senescence in hypoxia-induced PASMC proliferation, we analyzed the level of IL-6 in the culture supernatant by ELISA. We found that the level of IL-6 in cell supernatant in hypoxia group was significantly higher than that of normoxic group (*P* < 0.01). The results suggest that PASMC senescence significantly increased in hypoxia-induced PASMC proliferation; it was accompanied by up-regulation of IL-6 level in the culture supernatant, indicating that the senescence of PASMCs can promote cytokine IL-6 paracrine (Figure 3A and Table 1).

**TABLE 1:** The level of IL-6 in the culture supernatant in hypoxia-induced PASMC proliferation.

Groups	n (Multiple wells)	IL-6 (ng/mL)
Control	6	266.3 ± 23.56
Hypoxia	6	443.7 ± 54.299**
*P*		0.0047

**P < 0.01 vs. Control

### IL-6 Inhibitor, AX-024 Can Inhibit Cell Proliferation by Inhibiting Paracrine IL-6 in Hypoxia-Treated PASMCs

To further verify that PASMC senescence promoted PASMC proliferation by paracrine IL-6, AX-024 of IL-6 inhibitor was utilized. We found that the AX-024 with different concentration has different degree of inhibition against IL-6, and the inhibitor has the best inhibitory effect when the concentration of inhibitor was 10 nmol/mL (Figure 3B). The above result suggests that AX-024 of IL-6 inhibitors can significantly inhibit the paracrine of IL-6.

Then, the experiment was divided into normoxic group, hypoxia, and hypoxia + AX-024 group. ELISA was used to detect the paracrine level of IL-6 in the supernatant after AX-024 treatment. We found that the level of IL-6 in supernatant in the hypoxia + AX-024 group was significantly downregulated compared with that of the hypoxia group (*P* < 0.001) (Figure 3C and Table 2). The above results suggest that AX-024 of IL-6 inhibitor can inhibit the paracrine IL-6 in hypoxia-treated PASMCs.

**TABLE 2:** The level of IL-6 after AX-024 treatment in hypoxia-induced PASMCs proliferation.

Groups	n (Multiple wells)	IL-6 (ng/mL)
Control	6	510.4 ± 51.97
Hypoxia	6	731.8 ± 64.85*
AX-024	6	363.1 ± 49.61^##^

*P < 0.05; ^##^*P* < 0.01.

To clarify the proliferation inhibition after AX-024 treatment of IL-6 inhibitor, CCK8 was utilized to detect the proliferation of PASMCs. Western blot and qPCR were used to detect the expression of protein and mRNA of proliferation marker protein PCNA. We found that AX-024 of IL-6 inhibitor can significantly inhibit hypoxia-induced PASMC proliferation (*P* < 0.05) (Figure 3D). Western blot results showed that the protein expression of PCNA in hypoxia + AX-024 group was significantly lower than that in hypoxia group (*P* < 0.05) (Figure 3E). qPCR results showed that the mRNA expression of PCNA in hypoxia + AX-024 group was significantly inhibited compared with that of the hypoxia group (*P* < 0.05) (Figure 3F). The results indicated that AX-024 of IL-6 inhibitor can inhibit cell proliferation by inhibiting paracrine IL-6 in hypoxia-treated PASMCs.

### Rapamycin Can Inhibit PASMC Senescence by Inhibiting the Activation of mTOR/S6K1 Signaling in Hypoxia-Treated PASMCs

**FIGURE 4:**
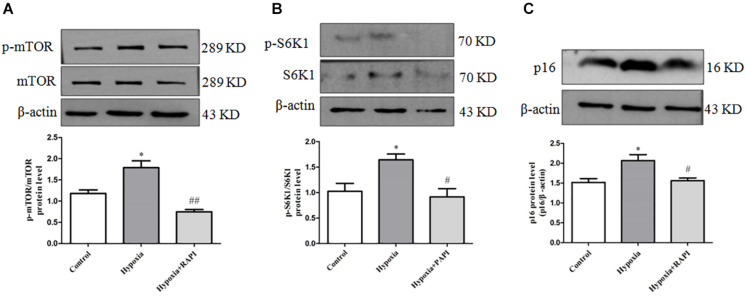
Rapamycin can inhibit PASMC senescence by inhibiting the activation of mTOR/S6K1 signaling in hypoxia-treated PASMCs. **(A)** The protein expression of p-mTOR and mTOR was determined by Western blot in PASMCs. **(B)** The protein expression of p-S6K1 and S6K1 was determined by Western blot in PASMCs. **(C)** The protein expression of p16 was determined by Western blot in PASMCs. Data are mean ± SEM, *n* = 3. ^∗^*P* < 0.05 vs. control, ^#^*P* < 0.05 vs. hypoxia, ^##^*P* < 0.01 vs. hypoxia.

In order to clarify the upstream regulation mechanism of PASMC senescence in hypoxia-treated PASMCs, rapamycin of mTOR inhibitor was utilized. We found that PASMCs cultured under hypoxia condition demonstrated a significant increase in phosphorylation of mTOR and S6K1 expression (Figures 4A,B). These data indicated that hypoxia can significantly induce mTOR and S6K1 activation in primarily cultured PASMCs, which may contribute to PASMC senescence, indicating that mTOR/S6K1 pathway was involved in PASMC senescence in hypoxia-induced PASMC proliferation.

To further confirm the key effect of mTOR/S6K1 pathway in hypoxia-induced PASMC proliferation, we used rapamycin of mTOR inhibitor in hypoxia-induced PASMC proliferation. We found that rapamycin, an inhibitor of mTOR, can strongly inhibit the activation of p-mTOR and p-S6K1 (Figures 4A,B). Importantly, we found that rapamycin could obviously suppress the expression of senescence-related protein P16 (Figure 4C), suggesting that mTOR promotes PASMC senescence in hypoxia-induced PASMC proliferation. Therefore, the above results indicate that rapamycin, an inhibitor of mTOR, could inhibit PASMC senescence in hypoxia-treated PASMCs.

### Rapamycin Can Inhibit Hypoxia-Induced PASMC Proliferation

**FIGURE 5:**
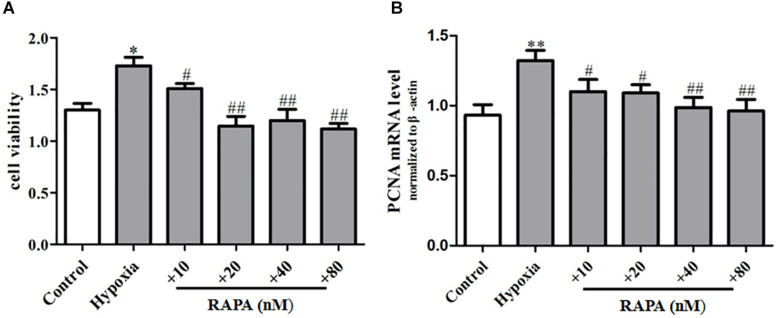
Rapamycin can inhibit hypoxia-induced PASMC proliferation. **(A)** The proliferation ability of PASMCs was measured by CCK8 assay. **(B)** The mRNA expression of proliferative marker protein PCNA was measured by qPCR. Control: cells were treated with 21% O_2_ for 48 h. Hypoxia: cells were treated with 3% O_2_ for 48 h; + RAPA 10–80 nM: cells were treated with RAPA 10–80 nM and then subjected to 3% O_2_ for 48 h, respectively. Data are mean ± SEM, *n* = 3. ^∗^*P* < 0.05, ^∗∗^*P* < 0.01, ^#^*P* < 0.05, ^##^*P* < 0.01 vs. hypoxia.

In order to further explore the regulation effect of mTOR on PASMC senescence, rapamycin was used to incubate PASMCs for 48 h, and then PASMC proliferation was analyzed by CCK8. We found that rapamycin could largely decrease the proliferation of PASMCs compared to that of the hypoxia group (Figure 5A) and also inhibit the mRNA expression of proliferation marker PCNA (Figure 5B), suggesting rapamycin, an inhibitor of mTOR, can suppress hypoxia-induced PASMC proliferation by inhibiting PASMC senescence.

### mTOR siRNA Can Inhibit PASMC Senescence by Inhibiting the Activation of mTOR/S6K1 Signaling in Hypoxia-Treated PASMCs

**FIGURE 6:**
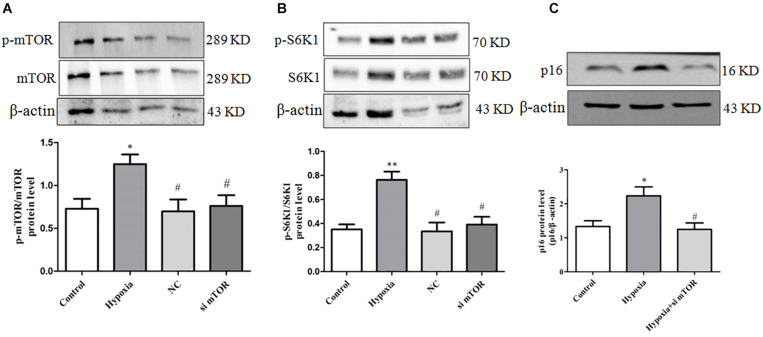
mTOR siRNA can inhibit PASMC senescence by inhibiting the activation of mTOR/S6K1 signaling in hypoxia-treated PASMCs. **(A)** mTOR siRNA decreased the level of mTOR protein; the protein expression of mTOR was determined by Western blot; statistical result of total gray value was estimated by the image analysis software. **(B)** mTOR siRNA decreased the level of p-S6K1 and S6K1; the protein expression of p-S6K1 and S6K1 was measured by Western blot; statistical result of total gray value was estimated by the image analysis software. Control: cells were treated with 21% O_2_ for 48 h; hypoxia: cells were treated with 3% O_2_ for 48 h; + NC: cells were transfected with negative control 50 nM for 4 h before stimulation with hypoxia for 48 h; + mTOR siRNA 50 nM: cells were transfected with mTOR siRNA 50 nM for 4 h before stimulation with hypoxia for 48 h. **(C)** mTOR siRNA decreased the protein expression of p16; the protein expression of p16 was determined by Western blot; statistical result of total gray value was estimated by the image analysis software. Data are mean ± SEM, *n* = 3. ^∗^*P* < 0.05 vs. control, ^∗∗^*P* < 0.01 vs. control, ^#^*P* < 0.05 vs. hypoxia.

To further confirm the key effect of mTOR/S6K1 pathway in hypoxia-induced PASMC proliferation, we used mTOR siRNA in hypoxia-induced PASMC proliferation. We found that mTOR siRNA can strongly inhibit the protein expression of mTOR and S6K1 (Figures 6A,B). Importantly, we found that mTOR siRNA could obviously suppress the expression of senescence-related protein P16 (Figure 6C), suggesting that mTOR promotes PASMC senescence in hypoxia-induced PASMC proliferation. The above results further support that mTOR could inhibit PASMC proliferation via inhibiting PASMC senescence.

### mTOR siRNA Can Inhibit Hypoxia-Induced PASMC Proliferation

**FIGURE 7:**
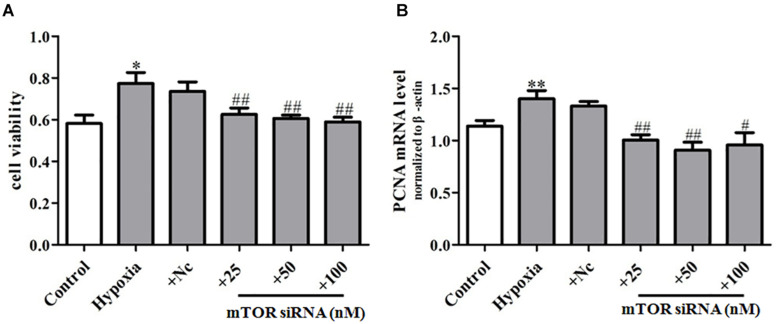
mTOR siRNA can inhibit hypoxia-induced PASMC proliferation. **(A)** Proliferation of PASMCs was measured by CCK8 assay. **(B)** The mRNA expression of PCNA was determined by qPCR. Control: cells were treated with 21% O_2_ for 48 h; hypoxia: cells were treated with 3% O_2_ for 48 h; + NC: cells were transfected with negative control 50 nM for 4 h before stimulation with hypoxia for 48 h; + si mTOR: cells were transfected with 25, 50, and 100 nM mTOR siRNA for 4 h before stimulation with hypoxia for 48 h, respectively. Data represent mean ± SEM. *n* = 3. ^∗^*P* < 0.05 vs. control, ^∗∗^*P* < 0.01 vs. control, ^#^*P* < 0.01 vs. hypoxia, ^##^*P* < 0.05 vs. hypoxia.

In order to further explore the regulation effect of mTOR on PASMC senescence, we transfected primary PASMCs with mTOR siRNA (25, 50, 100 nM), observing that mTOR siRNA suppressed markedly the proliferation of PASMCs in hypoxia-treated PASMCs (Figure 7A) and also inhibited the mRNA expression of proliferation marker PCNA (Figure 7B), compared with that of the hypoxia group, which suggest that mTOR siRNA could inhibit the proliferation of PASMCs through inhibiting the senescence of PASMCs. Taken together, these data demonstrated that mTOR/S6K1 signaling can promote PASMC senescence and in turn increase hypoxia-induced proliferation of PASMCs.

## Discussion

The pathogenesis of PH is very complex and is a pathophysiological process involving multiple factors. The occurrence of pulmonary vascular remodeling in PH involves a variety of cells (endothelial cell origin, mesangial cell origin, smooth muscle cell itself proliferation origin) (Deng et al., 2015; Ranchoux et al., 2015; Volz et al., 2015), cytokines, and inflammatory factors (IL-6, FGF-2, TGF-β, etc.) (Kim et al., 2015; Simpson et al., 2020) and involves a variety of pathways, such as TGF β, ET1, NO, FGF-2, BMP-ID pathway, TGF β/BMPs-Smad pathway (Abe et al., 2010; Dromparis et al., 2013; Bochenek et al., 2020; Hodgson et al., 2020). Studies have confirmed that PASMC proliferation plays an important role in the occurrence and development of pulmonary vascular remodeling in PH. However, what factors lead to PASMC proliferation and promote pulmonary vascular remodeling in PH are not fully understood. Our study reveals the pathophysiological mechanism of PH from a new perspective and discusses the important role of PASMC senescence as a new pathophysiological mechanism of PH, to explore the important role of PASMC senescence in hypoxia-induced PH and the mechanism of senescence PASMC in secretion of IL-6 promoting PASMC proliferation. On this basis, we elucidate that mTOR/S6K1 pathway can increase PASMC senescence and promote PASMC proliferation in HPH. It will have an exploratory experimental foundation for revealing the pathogenesis of PH and controlling the development of PH and expand a new idea for the prevention and treatment of PH.

Studies have confirmed that SASP is a secretion composed of a variety of proinflammatory factors, metalloproteinases, and growth factors (Dorr et al., 2013). The secretion of SASP can change the tissue microenvironment and lead to pathological changes of adjacent cells, thus promoting the progress of the disease (Koobatian et al., 2015). Recent clinical studies have found that PASMC senescence is significantly increased in patients with COPD, but the specific mechanism has not been elucidated (Noureddine et al., 2011). Our study results show that PASMC senescence may play an important role in the hypoxia-induced proliferation of PASMCs. In order to explore the role of PASMC senescence in hypoxia-induced proliferation of PASMCs, we used hypoxia-treated primary PASMCs, to detect the changes of β-galactosidase activity and the expression of p21 and p16 protein in hypoxia-induced PASMC proliferation. Our results showed that hypoxia can promote the number of β-galactosidase positive cells and the expression of senescence marker p21, p16 protein up-regulated in hypoxia-induced PASMCs. The above studies show that PASMC senescence is increased significantly in hypoxia-induced PASMC proliferation.

As hypoxia can promote PASMC senescence, what role does PASMC senescence play in hypoxia-induced PASMC proliferation? The study found that senescent cells produce a variety of cytokines (SASP), including IL-6, IL-8, TNF-α, and MCP-1, which promote the proliferation of adjacent cells in an autocrine or paracrine manner (Mistriotis and Andreadis, 2017). Previous studies have found that senescence aortic smooth muscle cells promote the development of atherosclerosis by secreting SASP (IL-1α) (Gardner et al., 2015). Noureddine et al. (2011) found that the IL-6 secretion of senescence PASMCs in COPD can regulate PASMC proliferation. Therefore, we speculate that IL-6 may be involved in the regulation of PASMC proliferation in hypoxia-treated PASMCs. To confirm whether IL-6 is involved in PASMC proliferation, first, the level of IL-1β, TGF-β, IL-6, IL-8, MCP-1, and TNF-α in hypoxia-induced PASMCs was screened. The results showed that the level of IL-6 in hypoxia-promoting PASMC senescence was significantly increased in hypoxia-induced PASMC proliferation. These results indicate that IL-6 may play a more important role than other cytokines in hypoxia-induced PASMC proliferation. Our results found that hypoxia promotes PASMC senescence at the same time; the level of IL-6 in the culture supernatant was up-regulated, suggesting that the senescence PASMCs can promote the secretion of cytokine IL-6 in hypoxia-induced PASMC proliferation. To further determine the role of IL-6 in hypoxia-induced PASMC proliferation, AX-024 of IL-6 inhibitors was used to block IL-6 secretion in hypoxia-induced PASMC proliferation. The results showed that the secretion of IL-6 in the supernatant of cells decreased significantly after blocking IL-6 secretion, and PASMC proliferation was significantly inhibited in hypoxia-treated PASMCs. Based on the above research, we suggest that senescence PASMCs promote the abnormal proliferation of PASMCs through secretion of IL-6 and promote the occurrence of PH.

As senescence of PASMCs plays an important role in hypoxia-induced PASMC proliferation, however, what factors (upstream pathways) affect PASMC senescence in hypoxia-induced PASMC proliferation are another important issue that we focus on. In order to clarify the upstream regulatory pathways of PASMC senescence in the process of PH, we reviewed some literature and conducted key preliminary experiments. Studies have shown that mTOR regulates the senescence of IMR90 cells by regulating SASP (Herranz et al., 2015). Christy et al. (2015) also found that the interaction between P53 and mTOR could affect the senescence of fibroblasts. In addition, Yang et al. (2014) reported that 14,15-epoxidated dicastrienoglyceric acid participated in the regulation of endothelial cell senescence via mTOR. These results suggest that mTOR may be involved in the regulation of vascular cells senescence. Our key preliminary results indicated that hypoxia promoted the senescence of PASMCs accompanied with increasing of p-mTOR levels. The above results suggest that mTOR may be involved in regulating the senescence of PASMCs. Rapamycin, an mTOR-specific inhibitor, was further used to treat PASMCs. The results showed that rapamycin significantly inhibited the increasing of PASMC senescence, reduced the protein level of p-mTOR, and then inhibited PASMC proliferation in hypoxia-induced PASMCs. After further treatment with mTOR siRNA in hypoxia-induced PASMC proliferation, mTOR siRNA could significantly inhibit the expression of mTOR, which in turn inhibited the expression of P16 and then inhibited hypoxia-induced PASMC proliferation. These results support that mTOR plays an important role in promoting PASMC senescence and thus promotes hypoxia-induced PASMC proliferation.

Ribosomal protein S6 kinase 1 (S6K1) is an important effector protein downstream of mTOR, which has Ser/Thr kinase activity and mediates protein synthesis, mRNA processing, cell growth, and survival (Um et al., 2004; Yuan et al., 2013). Studies have shown that abnormal mTORC1-S6K1 signaling pathway leads to numerous pathological changes, including diabetes, obesity, cancer, organ hypertrophy, and senescence-related diseases (Cornu et al., 2013). Studies have shown that down-regulation of the mTORC1-S6K1 signaling pathway can prolong the life cycle of mice (Liao et al., 2017). In addition, knockout of rps6KBL (encodes S6K1) in mice was discovered to extend the lifespan of female mice (Selman et al., 2009). In our study, we found that S6K1 is activated in hypoxia-promoted PASMC senescence, and rapamycin (the mTOR inhibitor) can inhibit the activation of S6K1, in turn inhibiting the senescence of PASMCs and then inhibiting the hypoxia-induced PASMC proliferation. mTOR siRNA also significantly inhibited the activation of S6K1, inhibited the PASMC senescence, and then inhibited hypoxia-induced PASMC proliferation, which was consistent with the effect of rapamycin. These results suggest that the mTOR/S6K1 pathway is involved in the regulation of PASMC senescence in hypoxia-induced PASMC proliferation and thereby promotes PASMC proliferation in PH.

Our results reveal PASMC senescence is a key role in hypoxia-induced PASMC proliferation. We found that PASMC senescence can promote hypoxia-induced PASMC proliferation through paracrine IL-6, and on this basis, we have clarified that mTOR/S6K1 pathway can accelerate PASMC senescence and in turn promote the PASMC proliferation in hypoxia-induced PASMCs. Our research will help to further reveal the pathogenesis of PH and provide a new target for seeking new drugs against PH.

## Data Availability Statement

The raw data supporting the conclusions of this article will be made available by the authors, without undue reservation.

## Ethics Statement

The animal study was reviewed and approved by the Laboratory Animal Center, University of South China. Written informed consent was obtained from the individual(s), and minor(s)’ legal guardian/next of kin, for the publication of any potentially identifiable images or data included in this article.

## Author Contributions

Z-SJ and A-PW contributed to the conception of the study. A-PW, YT, S-XG, and X-FM contributed significantly to analysis and manuscript preparation. A-PW, FY, and J-HS performed the data analyses and wrote the manuscript. Z-SJ, QG, and X-PQ helped perform the analysis with constructive discussions. FY, J-HS, and WC did all the experiments. FY did a lot of experiments. QG analyzed and arranged the experiment data and helped perform the analysis with constructive discussions. All authors contributed to the article and approved the submitted version.

## Conflict of Interest

The authors declare that the research was conducted in the absence of any commercial or financial relationships that could be construed as a potential conflict of interest.
